# Scale-Dependent Signal Identification in Low-Dimensional Subspace: Motor Imagery Task Classification

**DOI:** 10.1155/2016/7431012

**Published:** 2016-11-03

**Authors:** Qingshan She, Haitao Gan, Yuliang Ma, Zhizeng Luo, Tom Potter, Yingchun Zhang

**Affiliations:** ^1^Institute of Intelligent Control and Robotics, Hangzhou Dianzi University, Hangzhou, Zhejiang 310018, China; ^2^Department of Biomedical Engineering, University of Houston, Houston, TX 77204, USA; ^3^Guangdong Provincial Work Injury Rehabilitation Center, Guangzhou 510000, China

## Abstract

Motor imagery electroencephalography (EEG) has been successfully used in locomotor rehabilitation programs. While the noise-assisted multivariate empirical mode decomposition (NA-MEMD) algorithm has been utilized to extract task-specific frequency bands from all channels in the same scale as the intrinsic mode functions (IMFs), identifying and extracting the specific IMFs that contain significant information remain difficult. In this paper, a novel method has been developed to identify the information-bearing components in a low-dimensional subspace without prior knowledge. Our method trains a Gaussian mixture model (GMM) of the composite data, which is comprised of the IMFs from both the original signal and noise, by employing kernel spectral regression to reduce the dimension of the composite data. The informative IMFs are then discriminated using a GMM clustering algorithm, the common spatial pattern (CSP) approach is exploited to extract the task-related features from the reconstructed signals, and a support vector machine (SVM) is applied to the extracted features to recognize the classes of EEG signals during different motor imagery tasks. The effectiveness of the proposed method has been verified by both computer simulations and motor imagery EEG datasets.

## 1. Introduction

Many people throughout the world live with a variety of clinical conditions, including stroke, spinal trauma, cerebral palsy, and multiple sclerosis. Unfortunately, these conditions frequently present with motor deficits, which greatly reduce the quality of life for those affected. Mental practice with motor imagery (MI) is currently considered a promising additional treatment to improve motor functions [1]—repetitive cognitive training exercise, during which the patient imagines performing a task or body movement without actual physical activity, has been shown to modulate the cerebral perfusion and neural activity in specific brain regions [2]. Interestingly, it has been suggested that the combination of robot-assisted training devices and brain-controlled limb assistive technology may help to induce neural plasticity, resulting in motor function improvement [3]. Despite recording noninvasively and on the same time scale as the sensorimotor control of the brain, the high-dimensional EEG data used in MI exercises faces many challenges [4]. More specifically, these signals are usually collected from multiple electrodes (or channels), which are inevitably contaminated by the noise from biological, environmental, and instrumental origins.

Dimensionality reduction plays a key role in many fields of data analysis [5]. Using this method, data from a high-dimensional space can be represented by vectors in a reduced, low-dimensional space in order to simplify problems without degrading performance. One of the most popular dimensionality reduction methods is principle component analysis (PCA) [6], which is theoretically guaranteed to discover the dimensionality of the subspace and produce a compact representation if the data is embedded in a linear subspace. In many real world problems, however, there is no evidence that the data is actually sampled from a linear subspace [7, 8]. This has motivated researchers to consider manifold-based approaches for dimensionality reduction. Various manifold learning techniques, including ISOMAP, locally linear embedding (LLE), and Laplacian eigenmaps, have been proposed to reduce the dimensionality of fixed training sets in ways that maximally preserve certain interpoint relationships [9–11]. Unfortunately, these methods do not generally provide a functional mapping between the high- and low-dimensional spaces that is valid both on and off the training data [7]. Recently, spectral methods have also emerged as powerful tools for dimensionality reduction. Spectral regression (SR), based on regression and spectral graph analysis, can make efficient use of both labeled and unlabeled points to discover the intrinsic discriminant structure in the data [7, 8]. As a result, SR has been applied to supervised, semisupervised, and unsupervised situations across different pattern recognition tasks [12, 13] and has shown its superiority over traditional dimensional reduction methods.

Empirical mode decomposition (EMD) is a fully data-driven and adaptive analysis method that is widely applied within the field of biomedical signal processing [14–16]. It decomposes a raw signal into a set of intrinsic mode functions (IMFs) which represent the natural oscillatory modes contained within the original data. EMD does have some limitations in processing multichannel data, since the IMFs decomposed from different data channels are difficult to match in number and/or frequency [17, 18]. In order to resolve this problem, a noise-assisted multivariate EMD (NA-MEMD) [19] method has been proposed recently. This method applies the dyadic filter bank property of multivariate EMD [20] to white noise and is thereby capable of reducing the mode-mixing problem significantly, achieving favorable performance in the classification of MI EEG signals [21]. Although EMD and its extended versions have been widely researched and applied, there have been few studies on the selection of relevant IMF levels (scales), raising the question of how to select the information-bearing IMF components in an efficient way. Conventional approaches make use of prior knowledge in task-related domains: relevant IMFs are selected by calculating the average power spectra of the first several IMFs and comparing them to the frequency distributions of the* mu* (8–12 Hz) and* beta* rhythms (18–25 Hz) [21]. Similarly, in the neural beta-related oscillatory activities, the informative IMFs are chosen by examining the mean beta band frequency [22]. In [23], the relevant modes are selected by means of partial reconstruction and measures of similarity are calculated between the probability density function of the input signal and that of each mode extracted by EMD, though this is still insufficient to analyze multivariate data. Recently, a novel statistical approach has been proposed to recognize the information-bearing IMFs on each scale [24]. This method uses similarity measures to compare the IMFs to both the data and noise, yielding impressive results when applied to the multichannel local field potentials recorded from the cortices of monkeys during generalized flash suppressing (GFS) tasks.

In this work, we propose a novel method to identify the information-bearing components from EEG data in low-dimensional space, independent of prior knowledge. The proposed method first performs NA-MEMD on the input signal to obtain different scales of IMFs. Secondly, unsupervised kernel spectral regression is employed to map the decomposed IMFs into a low-dimensional subspace, avoiding the eigendecomposition of dense matrices and enabling the flexible incorporation of various regularizers into the regression framework [7, 8]. Thirdly, a Gaussian mixture model (GMM) is generated, informed by the IMFs from both the original signal and noise, and an optimal number of clusters and corresponding model parameters are estimated by the GMM clustering approach. Finally, the information-bearing IMFs from the input signal are discriminated on each scale. The GMM clustering algorithm is essentially similar to conventional clustering algorithms (e.g., *K*-means, performing a hard assignment of data points to clusters) except that it allows cluster parameters to be accurately estimated even when the clusters overlap substantially [25]. Compared to existing methods of identifying informative IMFs, the new method has several noteworthy aspects:

1. Kernel spectral regression is employed to reduce the dimension of the decomposed IMFs by constructing a nearest neighbor graph to model their intrinsic structure.
2. The probability density function of the composite IMFs is modeled by a mixture of Gaussian distributions and the number of clusters which best fits the composite IMFs is estimated and used to recognize the information-bearing components.
3. The method does not depend on prior knowledge and can discriminate the informative IMFs from each signal channel on each scale.

The rest of the paper is organized as follows: Section 2 presents the materials and proposed signal identification method, consisting of the noise-assisted multivariate empirical mode decomposition of multichannel EEG signals, the spectral regression-based dimensionality reduction of the composite data created by combining the IMFs from signal and noise channels, and GMM clustering. It then briefly introduces the common spatial patterns-based feature extraction of the reconstructed signals from the identified information-bearing IMFs and support vector machine (SVM) classifier.  Section 3 then demonstrates the experimental results, including simulation results and applications on real MI EEG datasets. Finally, we provide some concluding remarks and suggestions for future work in Section 4.

## 2. Materials and Methods

### 2.1. Subjects and Data Recording

In order to assess the proposed algorithm, the EEG data from nine subjects was obtained from two publicly available datasets. These datasets contain EEG signals recorded while subjects imagined limb movements, such as left/right hand or foot movements. They are described briefly as follows:

(1) BCI Competition IV Dataset I [26] was provided by the Berlin BCI group. EEG signals were recorded using 59 electrodes from four healthy participants (*a*, *b*, *f*, and *g*) who performed two classes of MI tasks. More precisely, subjects *a* and *f* performed left hand and foot MI while subjects *b* and *g* carried out left hand and right hand MI. A total of 200 trials were available for each subject, including 100 trials for each class.
(2) BCI Competition III Dataset IVa [27] was provided by the Berlin BCI group. EEG signals were recorded using 118 electrodes from five healthy subjects (*aa*, *al*, *av*, *ay*, and *aw*) who performed right hand and foot MI. A training set and a testing set were available for each subject, though their size differed for each subject. In total, 280 trials were available for each subject, among which 168, 224, 84, 56, and 28 trials comprised the respective training sets for subjects *aa*, *al*, *av*, *ay*, and *aw*, with the remaining trials belonging to their testing sets.

**Figure 1:**
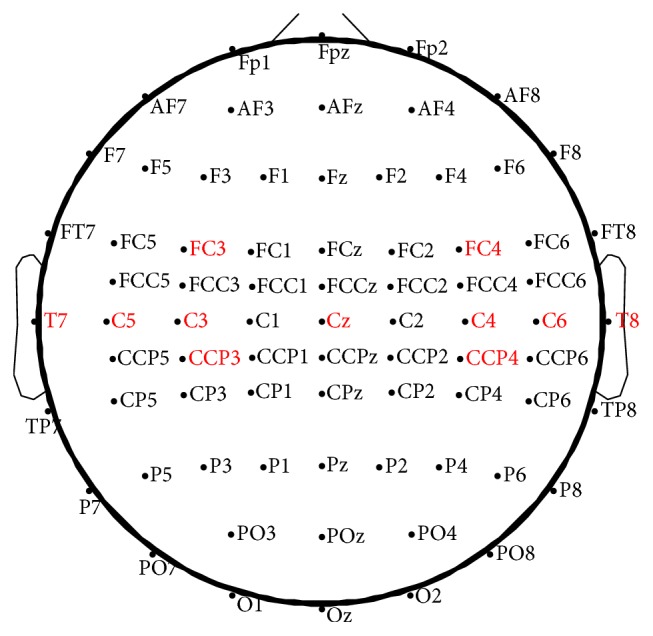
The positions of the chosen electrodes in the extended international 10–20 system.

Since the sensorimotor rhythms (SMRs) of motor imagery are primarily linked to the central area of the brain [28, 29], 11 EEG channels from the experimental data were used (FC3, FC4, Cz, C3, C4, C5, C6, T7, T8, CCP3, and CCP4, as recommended in [21]). The locations of these channels are shown in Figure 1.

### 2.2. Signal Identification in Low-Dimensional Subspace

Our goal is to identify the significant information-bearing IMFs on each scale for multichannel data. For each set of multivariate IMFs obtained by NA-MEMD, it is key to recognize the suitable IMFs bearing significant information associated with the MI EEG activities. In this section, we introduce a novel four-stage method to identify the informative IMFs. First, the NA-MEMD algorithm is performed on the original data to obtain a set of multivariate IMFs, from which the composite data is created by combining the IMFs from each signal channel with those from the noise channels on each scale. Secondly, the composite data is mapped into lower-dimensional subspace to extract feature vectors using unsupervised kernel spectral regression [7, 8]. Thirdly, a Gaussian mixture model is informed by exploiting the intrinsic discriminant structure of the probability distribution that generates the low-dimensional feature vectors. Then, for each group of feature vectors on each scale, the maximum likelihood classification is performed to distinguish them into classes after an optimal number of clusters and corresponding model parameters are estimated by the GMM clustering approach [25]. Finally, the informative IMFs from each signal channel on each scale are identified according to the clustering results. In the following sections, more details are provided for each stage of the proposed approach.

#### 2.2.1. Noise-Assisted Multivariate Empirical Mode Decomposition

For multivariate signals, the MEMD method [20] is utilized by generating multidimensional envelopes, taking signal projections along different directions, and finally averaging these projections to obtain the local mean. Though it is valid in processing multivariate nonstationary signals, MEMD still inherits a degree of mode-mixing. This has led to the recent development of the NA-MEMD approach [19], which is performed by adding white noise as additional channels in the original signal. NA-MEMD then enjoys both the benefits of the quasi-dyadic filter bank structure of MEMD on white noise and the additional realizations of white noise, guaranteeing the separability of the IMFs that correspond to both the original signal and noise. Given an *n*-variate input neuronal signal {**s**(*t*)}_*t*=1_^*L*^ = {*s*_1_(*t*), *s*_2_(*t*),…, *s*_*n*_(*t*)} with *L* samples per trial, MEMD produces *J* multivariate IMFs:

(1) $$\begin{array}{c} s\left(\;t\;\right)=\overset{J}{\underset{j=1}{\sum }}d_{j}\left(\;t\;\right)+r\left(\;t\;\right), \end{array}$$

where **d**_*j*_(*t*) denotes the *j*th IMF of **s**(*t*) and **r**(*t*) represents the *n*-variate residual.

In practice, the sifting process for a multivariate IMF can be stopped when all the projected signals fulfill a stoppage criterion. For MEMD sifting, a combination of EMD stoppage criteria is employed as introduced in [30, 31]. The stoppage criterion in standard EMD requires that the number of extrema and zero crossings differ at most by one for *τ* consecutive iterations in the sifting algorithm [30]. By introducing the envelope amplitude *ρ*(*t*) = 1/*κ*∑_*k*=1_^*κ*^|*e* ^*θ*_*k*_^(*t*) − *m*(*t*)| and defining an evaluation function *f*(*t*) = |*m*(*t*)/*ρ*(*t*)|, where *κ* denotes the total number of direction vectors in MEMD decomposition, *e* ^*θ*_*k*_^(*t*) represents the envelope curve along the *k*th (*k* = 1,…, *κ*) set of directions given by angles *θ*_*k*_ = {*θ*_1_^*k*^, *θ*_2_^*k*^,…, *θ*_*n*−1_^*k*^}, and *m*(*t*) is the local mean signal, another stoppage criterion is proposed [31]. The sifting process is continued until the value of *f*(*t*) is less than or equal to some predefined threshold *γ*. Similar to the given values in [20], *τ* = 5 and *γ* = 0.075 were chosen in this paper.

#### 2.2.2. Dimensionality Reduction by Spectral Regression

Spectral regression is an efficient method to reduce dimensionality from the graph embedding viewpoint [7, 8]. Specifically, an affinity graph is first constructed to learn the responses for labeled or unlabeled data and then the ordinary regression is applied for learning the embedding function. In essence, SR performs regression after the spectral analysis of the graph.

Suppose we have *N* data points {**x**_*i*_}_*i*=1_^*N*^ ⊂ *ℝ* ^*L*^, dimensionality reduction would aim to find a lower-dimensional representation {**z**_*i*_}_*i*=1_^*N*^ ⊂ *ℝ* ^*M*^, *M* ≪ *L*. Given a *p*-nearest neighbor graph *G* with *N* vertices, where the *i*th vertex corresponds to a data point **x**_*i*_, let **W** be a symmetric *N* × *N* matrix with *W*_*ij*_ having the weight of the edge joining vertices *i* and *j*. *G* and **W** can be defined to characterize certain statistical or geometric properties of the dataset.

Let **v** = [*v*_1_,…, *v*_*N*_]^T^ be the map from the graph to the real line, where T denotes a transposition. In the graph embedding approach [7], by introducing a linear function, *v*_*i*_ = *f*(**x**_*i*_) = **a** ^T^ **x**_*i*_, we find **X** ^T^ **a** = **v**, where **X** = [**x**_1_,…, **x**_*N*_] ∈ *ℝ* ^*L*×*N*^ and **a** = [*a*_1_,…, *a*_*N*_]^T^. The optimal embedding, **v**, is then given by the eigenvector corresponding to the maximum eigenvalue of the generalized eigenproblem

(2) $$\begin{array}{c} XWX^{T}a=\lambda XDX^{T}a \end{array}$$

with the eigenvalue *λ*, where **D** is a diagonal matrix whose entries are the column sums of **W**, *D*_*ii*_ = ∑_*j*_ *W*_*ji*_. This optimization can be solved through regression by adopting the regularization technique [7], and its solution is then given by

(3) a^=arg mina∑i=1NaTxi−vi2+α∑j=1Laj22+β∑j=1Laj,

where *v*_*i*_ is the *i*th element of **v**, the nonnegative regularization parameter *α* is used to control the amount of shrinkage, and some coefficients will be shrunk to exact zero if the nonnegative parameter *β* is large enough due to the nature of the *L*_1_ penalty. When the number of features is larger than the number of samples, the sample vectors will typically be linearly independent; thus the solutions to the optimization problem in (3) are the eigenvectors of the eigenproblem in (2) as *α* and *β* decrease to zero [7, 8]. The largest *M* eigenvectors of **a** are obtained according to the expected dimensionality of the reduced subspace in real applications. In this way, a low-dimensional representation of the sample matrix **X** is obtained as **Z** = **X** ^T^ **a**.

Similar to linear regression, by defining a nonlinear embedding function in reproducing kernel Hilbert space (RKHS), that is, *v* = *f*(**x**) = ∑_*i*=1_^*N*^ *a*_*i*_ *K*(**x**, **x**_*i*_) = *K*(**x**)^T^ **a**, where *K*(**x**, **x**_*i*_) is the Mercer kernel of RKHS and *K*(**x**) = [*K*(**x**, **x**_1_),…, *K*(**x**, **x**_*N*_)]^T^, the linear spectral regression approach can be generalized to kernel spectral regression (KSR) [8].

#### 2.2.3. Gaussian Mixture Model for Data Clustering

The Gaussian mixture model (GMM) is widely used as a probabilistic modeling approach to address unsupervised learning problems. Based on the expectation-maximization (EM) algorithm [32] and an agglomerative clustering strategy using Rissanen's minimum description length (MDL) criterion, a GMM-based clustering approach is developed [25]. The process begins with an initial number of clusters and a set of cluster parameters and iteratively combines the clusters until only one remains.

Let **Z** = [**z**_1_,…, **z**_*N*_] ∈ *ℝ* ^*M*×*N*^ be a set of *M*-dimensional samples belonging to different subclasses or clusters and let **y** = [*y*_1_,…, *y*_*N*_] be the subclass of each sample, where *y*_*i*_ ∈ {1,…, *c*} denotes which Gaussian distribution the sample **z**_*i*_ belongs to and *c* is the number of Gaussian components. The detailed steps of the GMM cluster algorithm are then given as follows.

(1) Initialize the parameters including the initial number of clusters *c*_*o*_ and the Gaussian model parameters **Ω** ^(0)^ = {{*π*_1_^(0)^, ***μ***_1_^(0)^, Σ_1_^(0)^},…, {*π*_*c*_^(0)^, ***μ***_*c*_^(0)^, Σ_*c*_^(0)^}}, where ***μ***_*k*_ is the mean vector, Σ_*k*_ is the covariance matrix for the *k*th Gaussian distribution, and *π*_*k*_ denotes the prior probability of the data point generated from the *k*th component, *k* = 1,…, *c*. The number of initial clusters in this case should be chosen to fit the number of data types for discrimination.

(2) Apply an iterative EM algorithm until the change in the MDL criterion (MDL(*K*, **Ω**)) is less than a threshold *ε*, where *ε* = 0.01 × (1 + *M* + (*M* + 1)*M*/2) × log⁡(*NM*):

(4) MDLc,Ω=−∑i=1Nlog⁡∑k=1cπkpzi ∣ yizi ∣ k,Ω+12υlog⁡NM,

where *p*_**z**_*i*_∣*y*_*i*__(**z**_*i*_∣*k*, **Ω**) is the Gaussian probability density function for the sample **z**_*i*_ given that *y*_*i*_ = *k*, log⁡(·) denotes the log-transformation and *υ* is the number of continuously valued real numbers required to specify the model parameters **Ω**, *υ* < 1/(2*NM*).

(3) Record the model parameter **Ω** ^(*c*,*i*_final_)^ and the value of the MDL(*c*, **Ω** ^(*c*,*i*_final_)^), where *i*_final_ denotes the final iteration of the EM updating process for each value of *c*.

(4) If the number of clusters is greater than 1, apply a defined distance function [25] to reduce the number of clusters, set *c* ← *c* − 1, and repeat Step (2).

(5) Choose the value $\hat{c}$ and the model parameters $\Omega^{\left(\hat{c},i_{final}\right)}$ which minimize the value of the MDL criterion.

(6) Based on the optimal parameters $\hat{c}$ and $\Omega^{\left(\hat{c},i_{final}\right)}$ from Step (5), sample vectors are distinguished into $\hat{c}$ classes using the maximum likelihood classification.

#### 2.2.4. Identification Algorithm for Information-Bearing IMFs

In this section, we introduce our algorithm for discriminating between informative and noninformative IMFs. The detailed steps of our method (KSR-GMM) are described as follows.

(1) Generate (*n* + *l*)-channel multivariate signal consisting of the input *n*-channel signal and an *l*-channel uncorrelated Gaussian white noise time-series of the same length as the input and then perform the MEMD decomposition [20] on the multivariate signal, obtaining (*n* + *l*)-variate IMFs denoted by (*n* + *l*) × *J* × *L* matrix, where *J* is the number of decomposition scales and *L* is the length of samples per channel.

(2) On the *j*th (*j* = 1,…, *J*) scale of the resulting multivariate IMFs from Step (1), combine the *l*-channel IMFs corresponding to the noise with the one-channel IMFs from the original signal, giving *n*-groups of (*l* + 1)-variate composite data given by *n* × (*l* + 1) × *L* matrix.

(3) At a given (*j*th) scale, the unsupervised KSR algorithm is performed, respectively, on the *i*th (*i* = 1,…, *n*) group of composite data obtained in Step (2), yielding *n*-groups of low-dimensional representation vectors denoted by *n* × (*l* + 1) × *M* matrix in the reduced subspace, where *M* is the number of reduced dimensions.

(4) At the given scale, for each group of representation vectors extracted in Step (3), the optimal number of clusters $\hat{c}$ is estimated by the GMM clustering approach and, based on the value of $\hat{c}$ and the corresponding model parameters, the representation vectors are then classified into $\hat{c}$ classes using the maximum likelihood classification.

(5) At the given scale, the information-bearing IMFs are identified according to the clustering results in Step (4): if an IMF from any individual signal channel is clustered with the IMFs from noise channels, then IMF is rejected as noninformative. All remaining IMFs are considered to be significantly information-bearing.

In this work, the initial number of clusters is chosen to be two in the GMM clustering, since we only discriminate two kinds of data: informative and noninformative IMFs. Additionally, it should be noted that excessive noise levels can compromise the data-driven ability of the NA-MEMD, though there is no technical limit on the number of the noise channels that can be added. As a rule of thumb, the variance of the noise is required to be within 2–10% of the variance of the input signal to produce reliable results [20].

### 2.3. Common Spatial Patterns for Feature Extraction

In the context of EEG signal processing, the common spatial patterns (CSP) approach aims at finding linear spatial filters that maximize the variance of EEG signals from one class while minimizing their variance from others [33]. Mathematically, the spatial filters are the stationary points of the following optimization problem:

(5) maxu Ju=uTE1E1TuuTE2E2Tu=uTC1uuTC2us.t. u2=1,

where **u** denotes a spatial filter, **E**_*i*_ represents the *n* × *L* data matrix from class *i* where *n* is the number of channels and *L* is the number of samples per channel, and **C**_*i*_ is the estimated spatial covariance matrix from class *i* ∈ [1,2]. Using the Lagrange multiplier method, the solution can be obtained as the eigenvectors of the generalized eigenvalue decomposition: **C**_1_ **u** = *ζ* **C**_2_ **u**, where *ζ* denotes the eigenvalue associated with **u**. The spatial filters are then the eigenvectors of **C**_2_^−1^ **C**_1_, which correspond to the largest and lowest eigenvalues.

With the projection matrix **U** = [**u**_1_,…, **u**_*n*_], the spatially filtered signal of a trial **E** is given as $\hat{S}=U^{T}E$. For discriminating between two classes of MI tasks, the extracted feature vectors are the logarithm of the spatially filtered signal:

(6) $$\begin{array}{c} f_{j}=\log\left(\;\frac{var\left({\hat{s}}_{j}\right)}{\sum_{i=1}^{2m}var\left({\hat{s}}_{i}\right)}\;\right), \end{array}$$

where ${\hat{s}}_{j}\;\;(j=1,\ldots ,2m)$ denotes the *m* first and last rows of $\hat{S}$ and the symbol var(·) denotes the variance.

### 2.4. Support Vector Machine Classification of MI EEG

The support vector machine (SVM) algorithm [34] is believed to be a state-of-the-art classification method due to its robustness to outliers and favorable generalization capability. The central idea of SVM is to separate data by finding the hyperplane that produces the largest possible margin, which is the distance between nearest data points of different classes.

The detailed steps of EEG processing are outlined as follows:

(1) Preprocess the *n*-channel EEG data using a 5th-order Butterworth filter, obtaining filtered data with the frequency band 8–30 Hz.
(2) Perform the proposed identification method on the composite signals which are acquired by combining an additional *l*-channel Gaussian white noise with the *n*-channel EEG data obtained in Step (1), identifying the information-bearing IMFs on each scale.
(3) For the *n*-channel EEG data, the informative IMFs distinguished from Step (2) are added together to construct the band-pass filtered signals.
(4) Process the reconstructed signals from Step (3) with the CSP algorithm to extract the feature vectors for different motor imagery tasks.
(5) Employ the SVM classifier to identify the classes of EEG during different MI tasks based on the extracted feature vectors in Step (4).

## 3. Experimental Results and Discussion

In this section, several experiments on simulated data and real world EEG data were performed to show the effectiveness of our proposed method. The new algorithm was constructed based on the spectral regression code (http://www.cad.zju.edu.cn/home/dengcai/Data/data.html) and the GMM clustering code found in the software package (https://engineering.purdue.edu/~bouman/software/cluster/). We used the LIBSVM toolbox [35] to implement the SVM classification of EEG data. For all methods using kernel applications, a Gaussian kernel function is chosen due to its validity and stability in experiments, that is, exp⁡(−‖**x**_*i*_ − **x**_*j*_‖^2^/2*η* ^2^), where the parameter *η* is the Gaussian kernel width. All the methods are implemented in MATLAB 2013a environment on a PC with a 2.5 GHz processor and 4.0 GB RAM.

### 3.1. Simulation Results

Our proposed method is first performed on the simulated data to verify its effectiveness. Unless otherwise specified, 15-channel noise data was generated using an uncorrelated Gaussian white noise time-series which has the same length as that of the input signal. Moreover, the variance of noise was set to be 6% of the variance of the input according to suggestions in [20]. Additionally, the number of nearest neighbors (*p* = 5) and the regularization parameters (*α* = 0.001 for *L*_2_ penalty and *β* = 0.01 for *L*_1_ penalty) were chosen by cross-validation in this simulation.

In this experiment, the same simulated data was generated as in [24]. A 3-channel synthetic signal [X(*t*), *Y*(*t*), *Z*(*t*)] with the length *N* = 1000 and the sampling rate *f*_*s*_ = 1000 Hz is

**Figure 2:**
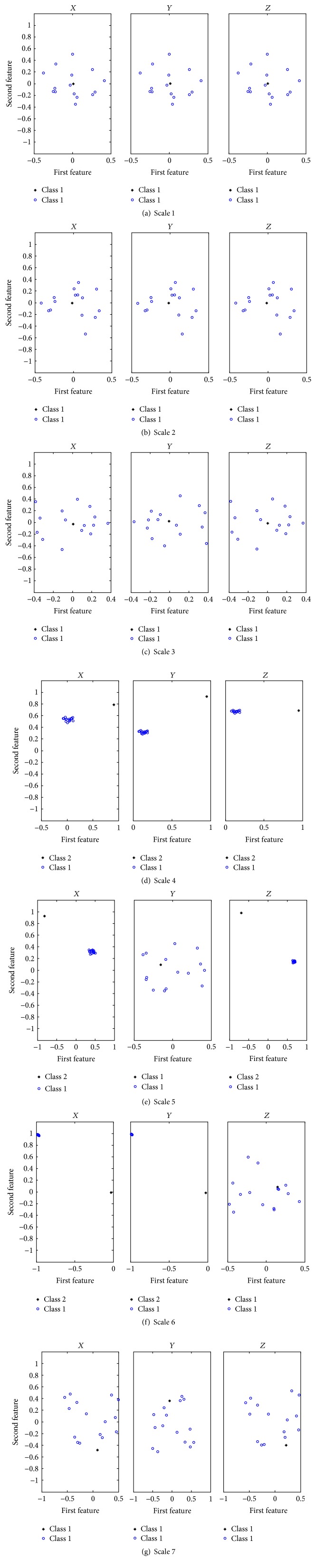
Clustering results using the proposed method at first seven scales.

(7) Xt=sin⁡2πf1t+sin⁡2πf2t+sin⁡2πf3t+q1t,t=1,2,…,1000Yt=sin⁡2πf1t+sin⁡2πf3t+q2t,t=1,2,…,1000Zt=sin⁡2πf2t+sin⁡2πf3t+q3t,t=1,2,…,1000,

where *f*_1_ = 12/*f*_*s*_, *f*_2_ = 26/*f*_*s*_, *f*_3_ = 50/*f*_*s*_, and *q*_1_(*t*), *q*_2_(*t*), *q*_3_(*t*) represent Gaussian white noises.

(I) To study the clustering performance of our method. A set of 3-channel input signals with SNR = 20 dB was generated and an additional 15-channel white noise with SNR = 6.1 dB was added to the input signal to create the composite signal. Our method was then performed on the composite signal and the information-bearing IMFs on each scale were identified.  Figure 2 shows a scatter plot with class labels of sixteen samples from a two-dimensional feature vector at the first seven scales, including one sample corresponding to one signal channel and fifteen samples from noise channels. Here, the data points corresponding to signal channels are represented by “*∗*” while those corresponding to noise channels are displayed by “o” in blue.

**Figure 3:**
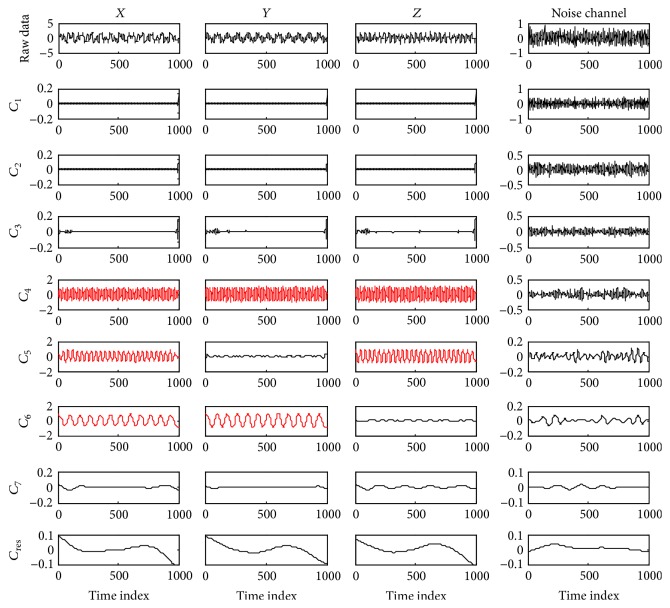
Decomposition results of the simulated data using the NA-MEMD algorithm.

It can be seen from Figure 2 that the composite data points on the 4th, 5th, and 6th scales in *X*-group are all clustered into two classes, with the same being true for the 4th and 6th scales in *Y*-group and the 4th and 5th scales in *Z*-group, while the composite data on the remaining scales of each channel falls into one class. According to the proposed method, these IMFs with two clusters are regarded as informative and the identification results are consistent with the IMFs containing the true frequency components decomposed by the NA-MEMD algorithm, as shown in Figure 3. The first seven IMFs are denoted as *C*_1_–*C*_7_ and the residuals are represented as *C*_res_, which are the sums of the remaining scales of IMFs. It can be seen that the underlying frequency components occur in the 4–6th IMF components, which are displayed in red.

(II) To test the effect of noise with different SNRs on our method, it was necessary to verify this performance since measured data often suffers from noise contamination in real applications. Our method was compared with several approaches for identifying information-bearing components: (i) Hu's method [24], which uses the Wasserstein distance to assess the similarity between the reference IMFs from noise channels and the IMFs from signal channels and subsequently establishes a confidence interval (e.g. 95%) for the distance by employing a Monte-Carlo technique, denoted as WD-CI; and (ii) three algorithms for dimensionality reduction together with GMM clustering: PCA, kernel PCA (KPCA) [36], and *L*_1_-norm PCA (L_1_PCA) [6]. In order to facilitate performance comparison, two kinds of error were evaluated. These are defined as (1) Type I error, which is the failure to identify true IMF components bearing relevant information, and (2) Type II error, which is the improper identification of information-free IMF components.

**Figure 4:**
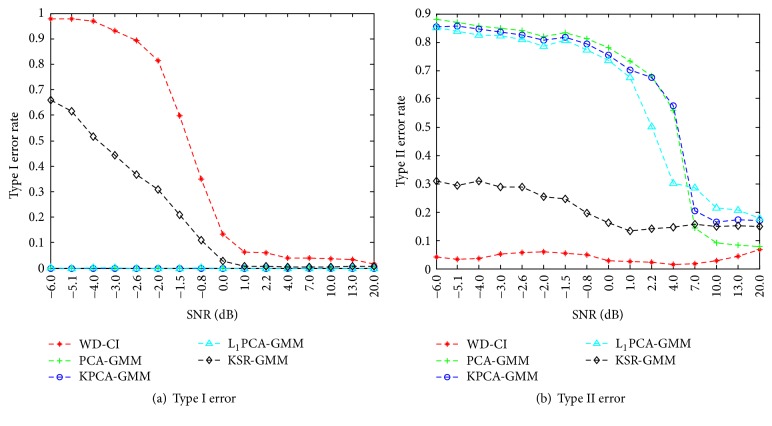
Statistical results by different methods at different SNRs which are systematically varied by changing the variance of the white noise superimposed in the signal.

First, different SNRs were varied by systematically changing the variance of the white noise superimposed in the input signal, combined with separate 15-channel white noise (SNR 6.1 dB) as reference channels. Overall, sixteen SNR levels were tested with 100 trials performed at each level. In each trial, the SNR of the white noise superimposed on the input signal was first changed, the relevant IMFs were identified by the different algorithms, and the corresponding error rates were calculated. The results from this test are shown in Figure 4. Low rates of Type I and Type II error were found at the higher SNR levels for all methods. On the whole, with the exception of Type I error rates in PCA-based approaches, increases in SNR led to decreases in error rates. When compared with other identification approaches, PCA-GMM, KPCA-GMM, and L_1_PCA-GMM showed lower Type I error rates but higher Type II error rates, while WD-CI yielded the lowest Type II error rate. The proposed method showed an improved Type I error rate with a slightly higher Type II error rate than the WD-CI algorithm, though the overall Type II error rates of both the new method and the WD-CI algorithm remain very small, even at low SNRs. These results indicate that our method is able to effectively identify the information-bearing components at low SNRs and is highly resistant to white noise.

**Figure 5:**
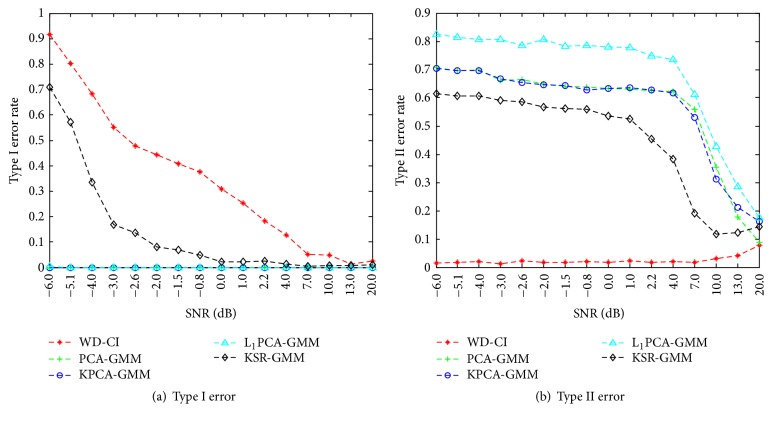
Statistical results achieved by different methods at different SNRs. SNRs were systematically varied by changing the variance of the red noise superimposed on the signal.

Next, considering that the noise contained in the signal channels is mismatched with the noise in the reference channels, the effects of red noises (1/*f* ^2^ noise) with different SNRs were tested on the proposed method.  Figure 5 shows the identification error rates at different noise SNR levels. Results indicate that both the new method and the WD-CI algorithm work well even when there is a mismatch between the noise contained in the data and the noise in the reference channels. This further demonstrates the robustness of our method when identifying the informative components in noisy data at low SNRs.

### 3.2. MI EEG Classification Results

This section evaluates the performance of our proposed method on MI EEG datasets. It has already been shown that the greatest result of motor imagery is a modulation of the SMRs [27]. Differential modulations in the SMRs were decomposed using the NA-MEMD method with locally orthogonal and narrowband IMF bases. Based on the identified information-bearing IMFs, relevant IMFs from the same channel were summed to get the reconstructed signal, and CSP-based feature extraction and SVM-based classification were performed.

For each trial in the BCI Competition IV Dataset I, we selected the EEG data from 0–4 s after the initiation of MI, as performed in [21]. In contrast, the window from 0.5–2.5 s after initiation was used for the BCI Competition III Dataset IVa, as in [37]. The 11-channel EEG data was regarded as the input signal and combined with an additional 15-channel noise (SNR 20 dB). Several parameters chosen by cross-validation in our identification algorithm are *p* = 5, *α* = 0.1, and *β* = 0. For both EEG datasets, the best model parameters were determined by fivefold cross-validation from {2^−10^,…, 2^10^} in SVM models. According to the aforementioned steps, experimental results are presented as the following.

(I) To demonstrate the identification capability of the informative IMF components in EEG data using the proposed method: it is noted that, for EEG data, unlike the simulations, we do not know the ground truth of the IMFs that have been identified. For all 200 trials of each subject in the BCI Competition IV Dataset I, the average power spectra of the identified information-bearing IMFs were computed and then compared to those obtained using the existing method (NA-MEMD-PK) [21].

**Figure 6:**
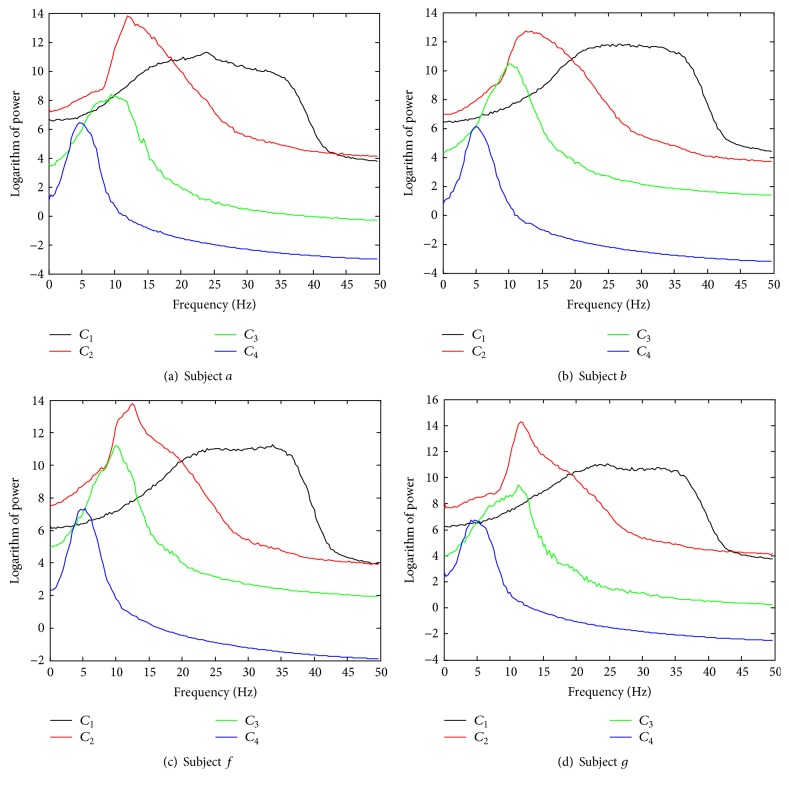
The average power spectra of *C*_1_ ~ *C*_4_ for all four subjects in BCI Competition IV Dataset I. Note that our method computes the average power spectra from the identified information-bearing IMFs at the first four scales for all 200 trials of each subject.

Figure 6 shows the logarithm of average power spectra for each subject using the new method. It can be seen that the* beta* and* mu* rhythms, which are contained in the 2nd (*C*_2_) and 3rd IMFs (*C*_3_), respectively, are separated clearly. Moreover, the frequency bandwidths in the 1st IMFs (*C*_1_) are generally broad, containing some parts of the 15–30 Hz frequency band. Consequently, there is a trade-off in the choice of *C*_1_; ignoring it would sacrifice some useful information, whereas conserving it could introduce noise. To resolve this problem, the role of the first scale is decided according to the optimal classification results combined with CSP-based feature extraction. For all four subjects, a paired *t*-test revealed no significant differences between the two approaches in the power spectra of all 200 trials at the first three IMFs but found a significant difference at the 4th IMF, as shown in Table 1. This demonstrates the validity of the proposed approach when identifying information-bearing IMFs from real EEG data.

(II) An evaluation of the classification performance of the proposed method using a fivefold cross-validation study on two MI datasets: the classification process here was repeated 100 times using the new method, the NA-MEMD-PK algorithm [21], and the non-EMD based approach in which raw data is directly processed by CSP-based feature extraction and SVM-based classification for a varying number of spatial filters (*m* = 1,2, 3,4). The average accuracy and standard deviation were obtained for each method and used for direct comparison.

**Figure 7:**
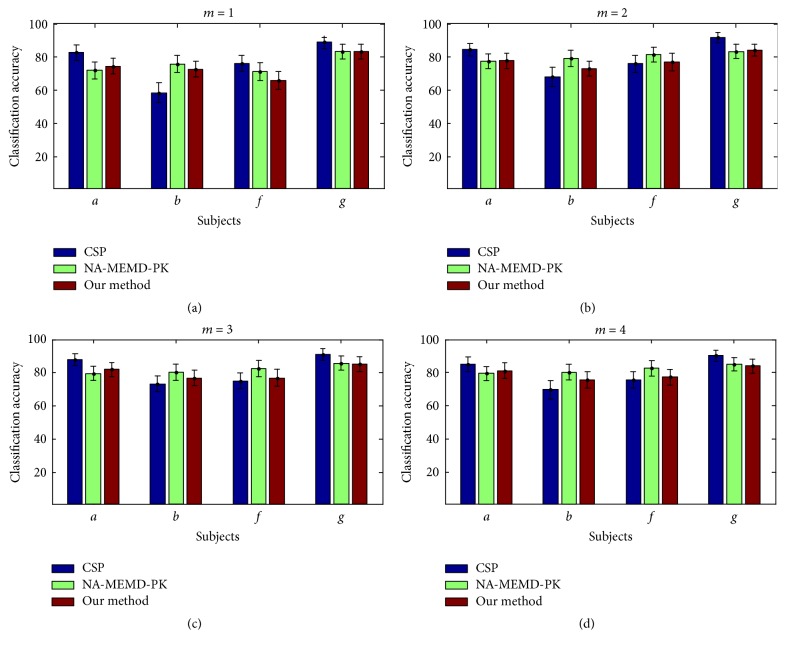
Classification accuracies (mean and standard deviation) obtained for the four subjects of BCI Competition IV Dataset I when *m* = 1,2, 3,4, respectively.

Considering the size of the total data for each subject in BCI Competition IV Dataset I, the number of EEG blocks was set at 140 for each training set and 60 for each testing set, as in [21]. To ensure a valid comparison between the different methods, the same data partitions were used in cross-validation.  Figure 7 shows the classification performances for all four subjects from the BCI Competition IV Dataset I. The results show that the NA-MEMD-PK approach yielded the best averaged results, with an average classification accuracy of 81.01% for all four subjects—a 0.24% improvement over the CSP algorithm and a 1.81% improvement over the new method. The CSP method yielded the best performance among the three approaches in two subjects (*a* and *g*), whereas NA-MEMD-PK yielded the best mean accuracy in the two remaining subjects (*b* and *f*), while our method performed slightly higher than the CSP algorithm when *m* = 2, 3. Nevertheless, a paired *t*-test revealed no significant difference between our method and the NA-MEMD-PK algorithm (*p* = 0.195, 0.096 for *m* = 2, 3, resp.), no significant difference between our method and the CSP approach (*p* = 0.074 when *m* = 2), and a significant difference between our method and the CSP approach (*p* = 0.003 for *m* = 3). These results show that, when compared to the NA-MEMD-PK algorithm, our method can achieve similar results without the use of prior knowledge.

**Figure 8:**
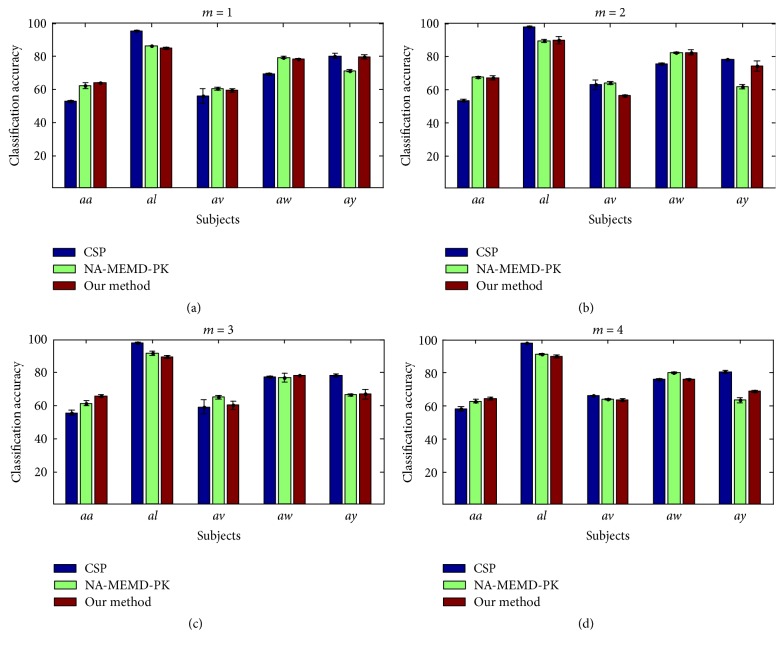
Classification accuracies (mean and standard deviation) obtained for the five subjects of BCI Competition III Dataset IVa when *m* = 1,2, 3,4, respectively.

Finally, the classification performances for the five subjects from the BCI Competition III Dataset IVa are demonstrated. For each subject, the CSP filters and classifier models were trained on the available training sets.  Figure 8 illustrates the classification accuracies (mean and standard deviation) obtained from these sets. The results showed that the average classification accuracy for all five subjects obtained by our method was 74.06%, yielding a 0.94% improvement over the NA-MEMD-PK approach. A paired *t*-test revealed no significant difference between our method and the NA-MEMD-PK algorithm (*p* = 0.225, 0.027 for *m* = 2, 3, resp.),and a significant difference between our method and the CSP approach (*p* values less than 0.01). When applied to the BCI Competition III data, the CSP method yielded the best performance among the three approaches in two subjects (*al* and *ay*), while the proposed algorithm performed the best in subject *aa* when *m* = 1,2, 3,4. Additionally, our method outperformed the NA-MEMD-PK approach in two subjects (*aa* and *ay*), whereas the NA-MEMD-PK algorithm performed better in two subjects (*al* and *av*) and yielded similar performance in subject *aw* for all four groups of spatial filters.

### 3.3. Discussion

In these experiments, the NA-MEMD algorithm exhibited an accurate localization of the task-specific frequency bands with favorable separability for feature extraction and classification, as demonstrated in its applications to MI EEG data. For the simulations, the new method was further shown to be robust to white and colored noises with different SNRs. When compared with other identification approaches (WD-CI, PCA-GMM, KPCA-GMM, and L_1_PCA-GMM), the proposed method obtained relatively improved performances in terms of both Type I and Type II error rates. For real EEG data, the information-bearing IMFs were discriminated clearly for nine subjects during MI tasks. When compared with the NA-MEMD-PK approach, which selects IMFs based on average power spectra, the proposed method yielded similar classification performance though it did not require prior knowledge to achieve such favorable results. Despite the favorable capability of the new algorithm when distinguishing the informative IMFs containing task-related frequency bands and classifying MI EEG signals, it should be recognized that individual subject differences may still have a great deal of influence on the recognition ability of the algorithm.

## 4. Conclusions

In this paper, we have shown how to discriminate the information-bearing components of motor imagery (MI) EEG independent of prior knowledge. The noise-assisted MEMD (NA-MEMD) algorithm was first performed on original datasets to obtain a set of multivariate IMFs, with the subsequent application of unsupervised kernel spectral regression (KSR) to generate low-dimensional feature vectors by mapping the decomposed IMFs into lower-dimensional subspace. For the low-dimensional feature vectors from each signal channel, a Gaussian mixture model (GMM) clustering approach was employed to estimate the optimal number of clusters and corresponding model parameters and then identify the information-bearing IMFs. The common spatial pattern (CSP) approach was exploited to train spatial filters to extract the task-related features from the reconstructed signals by adding the informative IMFs together. A support vector machine (SVM) classifier was applied to the extracted features and recognized the classes of EEG signals during different MI tasks. Using these techniques, we have demonstrated that our proposed method is effective at identifying the information-bearing IMF components in simulated data and MI EEG datasets and achieves excellent classification performance.

In conclusion, a novel method for scale-dependent signal identification in a low-dimensional subspace has been proposed for MI task classification. Although our method is independent of prior knowledge, entirely data-driven, and robust to different types of noise, several questions remain to be investigated in future work; the spectral regression-based dimensionality reduction approach selects the nearest neighbor graph; however this is not the only natural choice. Recently there has been a great deal of interest in exploring the different ways to construct a graph to model the intrinsic geometrical and discriminant structures within EEG datasets [38]. In addition, semisupervised clustering methods [39] have also yielded promising results when compared with the traditional unsupervised clustering approaches. To improve the clustering performance, it will be necessary to exploit the underlying manifold structure of the data along with additional knowledge from unlabeled data. Advancements such as these, in conjunction with the algorithm presented in this paper, will serve to improve the detection, classification, and evaluation of MI signals. This, in turn, can lead to improvements in EEG-based rehabilitation technologies, improving both the prediction and elicitation of motor recovery in a multitude of diseases worldwide [40].

## Figures and Tables

**Table 1:** *p* values comparing the power spectra of the first four IMFs identified by two approaches.

Subjects	1st IMFs	2nd IMFs	3rd IMFs	4th IMFs
*a*	0.581	0.995	0.536	0.007
*b*	0.899	0.989	0.866	0.004
*f*	0.656	0.998	0.958	0.030
*g*	0.540	1.000	0.777	0.010
